# Microfluidic Synthesis of Actuating Microparticles from a Thiol-Ene Based Main-Chain Liquid Crystalline Elastomer

**DOI:** 10.3390/polym8120410

**Published:** 2016-11-25

**Authors:** Tristan Hessberger, Lukas Braun, Rudolf Zentel

**Affiliations:** Department of Organic Chemistry, Johannes Gutenberg-University Mainz, Duesbergweg 10-14, 55099 Mainz, Germany; hessberger@uni-mainz.de (T.H.); l.braun@uni-mainz.de (L.B.)

**Keywords:** microfluidic, microparticles, liquid crystal, stimuli-responsive, soft actuator, thiol-ene, liquid crystalline elastomer, photo polymerization, continuous flow synthesis

## Abstract

In this article the microfluidic synthesis of strongly actuating particles on the basis of a liquid crystalline main-chain elastomer is presented. The synthesis is carried out in a capillary-based co-flow microreactor by photo-initiated thiol-ene click chemistry of a liquid crystalline monomer mixture. These microparticles exhibit a deformation from a spherical to a rod-like shape during the thermal-initiated phase transition of the liquid crystalline elastomer (LCE) at which the particles’ aspect ratio is almost doubled. Repeated contraction cycles confirm the complete reversibility of the particles’ actuation properties. The transition temperature of the LCE, the temperature range of the actuation process as well as the magnitude of the particles’ aspect ratio change are studied and controlled by the systematic variation of the liquid crystalline crosslinker content in the monomer mixture. Especially the variable actuation properties of these stimuli-responsive microparticles enable the possibility of an application as soft actuators or sensors.

## 1. Introduction

Liquid crystalline elastomers (LCEs) have been intensely studied and enhanced during the last decades with particular regard to their unique mesophasic behavior and elasto-mechanical properties [1,2,3,4,5,6]. LCEs are slightly crosslinked liquid crystalline polymer networks, which are capable of performing a reversible macroscopic shape change during the phase transition of the liquid crystalline state. The shape anisotropic molecules of a liquid crystalline phase (mesogens) enable the molecular self-organization into mesophases, which exhibit certain grades of molecular order. Nematic liquid crystals consist of rod-shaped (calamitic) mesogens, which are aligned parallel to a common director in the nematic phase. The deviation of the calamitic mesogens’ orientation from the director orientation is expressed by the nematic order parameter, which equals 1 for the ideal crystalline state and 0 for the isotropic state. Typical values of the order parameter for a nematic phase are between 0.3 and 0.7. In nematic LCEs, the parallel-aligned order of the mesogens is combined with the entropy-elastic properties of polymeric elastomers. The mesogens can either be linked to the polymer backbone via an alkyl spacer (side-chain LCE) or can be directly integrated into the polymer backbone (main-chain LCE). In the nematic phase, the uniform alignment of the mesogens forces the polymer chain into a deformed conformation (oblate or prolate). Heating above the clearing temperature causes the phase transition of the LCE from the nematic to the isotropic state at which the mesogen’s uniform alignment along the nematic director vanishes and a random coil conformation of the polymer chain is preferred [7].

The interplay between the polymer network and the nematic ordering of the mesogens may also modify the phase transition between the nematic and the isotropic phase. At first, this phase transition is “weakly first-order” in low molar mass nematics. That implies the existence of strong pretransitional effects in the isotropic phase (e.g., a strong Kerr effect resulting from the interaction of local nematic ordering with the external electric field). In LCEs, the polymer network can thus induce some orientation already in the isotropic phase (para-nematic phase). For strong mechanical fields this can change the phase transition between the nematic and the isotropic phase to become second-order. Secondly—and independent of the first effect—a broadened, continuous phase transition may also result from inhomogeneous crosslinking densities and different local clearing temperatures in the LCE sample [8,9,10].

Different external stimuli are known to trigger the phase transition and, therefore, a macroscopic shape change of the LCE. Depending on the chemical structure and composition of the mesogens, heating [11,12,13], ultraviolet (UV) irradiation [14,15,16] or solvent swelling [17] can initiate the phase transition of a stimuli-responsive liquid crystalline elastomer. The shape change of an oriented LCE as a response to an external stimulus offers the possibility for its application as actuators, sensors or microelectromechanical systems (MEMSs). Recent developments of utilizing the actuating properties of LCEs in technical applications range from a bio-inspired iris-like tunable aperture [18] and flow-controlling microvalves [19] to light-driven plastic motors [20].

The prerequisite for a macroscopic deformation of LCEs during a phase transition is an overall orientation of the mesogens along a common director, which leads to uniformly aligned liquid crystalline domains over the whole sample. Different orientation techniques are well established for the preparation of different LCE-based materials, such as mechanical stretching [2,21], electric [22] or magnetic fields [23] and surface forces [14]. Microfluidic devices offer another promising orientation method for liquid crystalline elastomers, in which the orientation process occurs mechanically in the flow field of a moving fluid. A UV-initiated on-the-fly synthesis of strongly actuating LCE microparticles with a narrow size distribution using a capillary-based microfluidic setup has been developed for different polymeric compositions of LCEs [15,24,25]. LCE actuators of various morphologies, such as spherical-, oblate- or rod-like-shaped particles [17], Janus particles [26], core-shell particles [24] and actuating fibers [25], are accessible. These architectures can be synthesized in a wide range of different sizes, combined with a fast production speed within the range of several minutes, as the orientation process, polymerization and crosslinking occur in one step. Furthermore, the precise control of the microfluidic setup parameters enables different director fields of the LCE orientation, which features different actuation patterns and geometries [25]. Similar microfluidic setups have been used to produce different kinds of microparticles from various polymeric materials [27,28,29].

The investigation and improvement of LCE materials in terms of their shape-changing properties (such as the magnitude of actuation, temperature range and reversibility) are still important for the development and optimization of new actuator applications. Especially main-chain elastomers are very attractive as actuators with regard to the strong coupling of the mesogens with the polymer backbone. Common synthetic pathways for main-chain LCEs are the crosslinking of liquid crystalline (LC) main-chain polymers prepared by poly-condensation and poly-addition reactions. For such LCEs, contractions of up to 500% have been reported [30,31]. However, the long reaction times needed for their synthesis represent a limiting factor for some orientation techniques, such as microfluidic devices, in which the polymerization and crosslinking process is typically initiated by UV-induced radical formation. Thiol-ene click chemistry provides an alternative synthesis route for main-chain LCEs [32]. The radical initiated addition of thiol groups to double bonds features the necessary reaction speed for fast processing in microfluidic devices. Such thiol-ene–mediated polymerizations have been used successfully for the preparation of linear polymers [33] and—In one case—For actuating main-chain LCEs [34].

In this article, the synthesis of main-chain LCE microactuators based on a thiol-ene click reaction is presented. Previous works by Fleischmann et al. [35] already demonstrated the possibility of producing actuating particles in a microfluidic device from a monomer mixture which contained a bifunctional liquid crystalline thiol-ene monomer and two non-mesogenic crosslinkers. However, the relative length changes of these actuators upon the phase transition reached merely 25%. Moreover, the liquid crystalline phase was unstable with regard to heating and a solvent had to be added in order to reduce the monomer mixture’s viscosity for the microfluidic processing. The main challenge of this study was therefore to investigate if a recently described monomer system for the preparation of main-chain LCEs can be adopted for microfluidic procedures and used successfully for the production of strongly actuating microparticles. Thus, a monomer mixture containing a liquid crystalline diacrylate crosslinker in addition to a diallyl and a dithiol monomer is used for the first time in microfluidics. It has been presented recently by White et al. for the preparation of programmed shape-changing LCE films. [34] By precise adjustment of the microfluidic setup, this monomer mixture provides a stable nematic phase during the polymerization process, yielding strongly actuating microactuators with changes of the particles’ aspect ratio almost up to 100% during the phase transition. Furthermore, the temperature range as well as the magnitude of the actuation process can be controlled by variation of the monomer mixture’s composition, featuring a microfluidic particle synthesis that provides microactuators with variable actuating properties.

## 2. Materials and Methods

### 2.1. Reagents and Materials

The liquid crystalline monomer 2-methyl-1,4-phenylene bis(4-(3-(allyloxy)propoxy)benzoate) was synthesized by a standard Steglich esterification [36] of 4-(3-(allyloxy)propoxy)benzoic acid with 2-methylbenzene-1,4-diol. The 4-(3-(allyloxy)propoxy)benzoic acid was synthesized as described in the literature [37]. The liquid crystalline crosslinker 2-methyl-1,4-phenylene bis(4-((6-(acryloyloxy)hexyl)oxy)benzoate) was synthesized by a standard Steglich esterification [36] of 4-((6-(acryloyloxy)hexyl)oxy)benzoic acid with 2-methylbenzene-1,4-diol. The 4-((6-(acryloyloxy)hexyl)oxy)benzoic acid was synthesized as described in the literature [38]. The UV-photoinitiator diphenyl(2,4,6-trimethyl-benzoyl)phosphine oxide (Lucerin TPO), the silicone oil (1000 cSt) and 1,2-ethanedithiol (EDT) were purchased from Aldrich (Sigma-Aldrich Chemie GmbH, Taufkirchen, Germany) and used as received.

The liquid crystalline monomer mixture was prepared for the microfluidic synthesis by dissolving equimolar amounts of the LC-monomer and EDT in dichloromethane. Then 20 to 60 mol % (referring to the LC-monomer) of the LC-crosslinker and 3 wt % of the photoinitiator were added to the solution. After complete solvation the dichloromethane was removed in a rotary evaporator at 10 mbar for 30 min. The resulting mixture was heated up to 110 °C in a pear shape flask for 10 min. The hot monomer mixture was then drawn in a 1 mL syringe.

### 2.2. Microfluidic Setup

**Figure 1:**
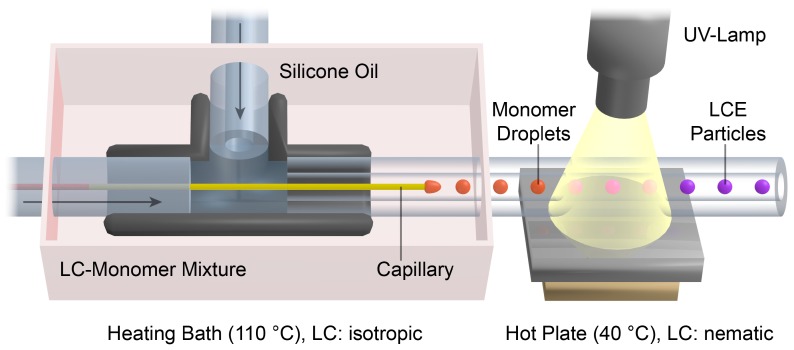
Schematic illustration of the co-flow microfluidic setup. The capillary displayed in yellow is connected to the PTFE (polytetrafluoroethylene) tube on the left side of the T-junction and provides the liquid crystalline monomer mixture. The continuous silicone oil phase is injected into the T-junction via the PTFE tube on the upper side. Monomer droplets are formed at the capillary’s tip and further polymerized by transportation through UV-light. (UV: ultraviolet, LC: liquid crystal, LCE: liquid crystalline elastomer).

The setup of the capillary based co-flow microreactor [17,39,40] is illustrated in Figure 1. The 1 mL syringe filled with the LC-monomer mixture was connected to the T-junction (ID: 1.25 mm) by a PTFE (polytetrafluoroethylene) microtube (ID: 0.17 mm). A second syringe (12 mL) was filled with the silicone oil (1000 cSt) and joined to the vertical plug of the T-junction via a PTFE microtube (ID: 0.75 mm). The glass capillary (ID: 100 µm, OD: 165 µm) was inserted into the PTFE tube of the monomer mixture. At the opposite plug of the T-junction the PTFE polymerization tube (ID: 0.75 mm) was placed around the tip of the glass capillary. The T-junction including the glass capillary was placed in an oil bath and tempered to 110 °C. Two syringe pumps (Harvard Apparatus Model 33 Twin Syringe Pump, Instech Laboratories, Plymouth Meeting, PA, USA) were used for the continuous injection of the LC-monomer mixture (flow rate: 0.05 mL/h) and the silicone oil (flow rates: 1.5 to 2.3 mL/h). The polymerization tube was passed over a hot plate (40 °C) and 7 cm were irradiated by UV-light using an Oriel LSH302 (500 W) lamp equipped with a band filter (323–385 nm) and a waveguide. At the end of the polymerization tube, LCE particles were collected in a snap-cap bottle.

### 2.3. Particle Analysis

The length measurements of the LCE-particles were carried out via polarized optical microscopy (Olympus BX51, Olympus Deutschland GmbH, Hamburg, Germany) in the temperature range of 30 to 250 °C. The heating of the particles was performed on a microscope hot-stage (Linkam TMS 94, Linkam Scientific Instruments, Waterfield, Tadworth, UK). Particles were placed on a glass microscope slide, embedded in a silicone oil (1000 cSt) matrix and heated at a heat rate of 30 °C/min. Thus, it took about 7 min and 20 s to measure one heating curve. Since the LCEs show an immediate response to temperature changes (less than a second), the particle’s length was measured during the heating. Particle images were taken under crossed polarizers using a microscope camera (Olympus DP22, Olympus Deutschland GmbH, Hamburg, Germany) and analyzed with a microscope imaging software (Olympus CellSens Standard V1.15, Olympus Deutschland GmbH, Hamburg, Germany).

## 3. Results and Discussion

### 3.1. Liquid Crystalline Monomer Mixture

**Figure 2:**
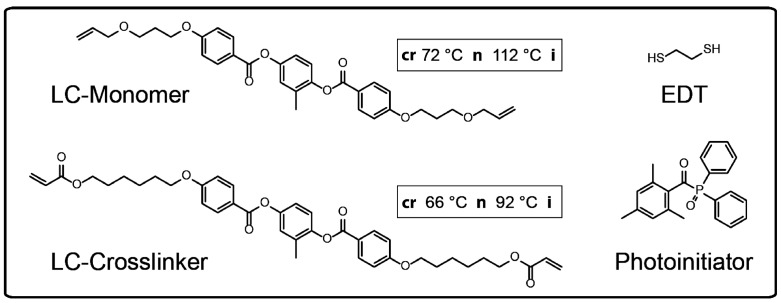
Chemical structures of the components which were used in the liquid crystalline monomer mixture. The phase transition temperatures of the mesogenic LC-monomer and LC-crosslinker were measured via differential scanning calorimetry. (EDT: 1,2-ethanedithiol).

For the synthesis of actuating LCE particles, a liquid crystalline monomer mixture was used, which was utilized for the preparation of cast films by White et al. [34]. Figure 2 illustrates the components of the LC-monomer mixture. The amount of the diacrylate LC-crosslinker was varied in the range of 20 to 60 mol % with respect to the LC-monomer amount. The LC-monomer and the LC-crosslinker were synthesized in our laboratory and the phase behavior was characterized by differential scanning calorimetry (DSC), in which a nematic phase of the LC-monomer (crystalline 72 °C nematic 112 °C isotropic) and a nematic phase of the LC-crosslinker (crystalline 66 °C nematic 92 °C isotropic) were observed. The mesomorphic phase behavior of the LC-monomer mixture depends on the molar parts of the LC-crosslinker moiety. A nematic phase with clearing temperatures in the range of 60 to 83 °C (at molar parts of the LC-crosslinker from 20 to 60 mol %) occurs by cooling down the monomer mixture from the isotropic phase [34].

### 3.2. Microfluidic Preparation

Actuating LCE particles should be fabricated in a continuous flow (co-flow) microfluidic reactor. A schematic illustration of the microreactor and the droplet formation and polymerization process are displayed in Figure 1.

The basic principle of the microfluidic particle synthesis is the dispersion of a liquid monomer mixture (dispersed phase) in a continuously flowing, highly viscous silicone oil (continuous phase). The droplet formation occurs at the tip of a thin glass capillary, which is placed in the middle of a PTFE tube (polymerization tube). The monomer mixture is pumped through the glass capillary and the surrounding continuous flow of the silicone oil causes a shear strain on the developing monomer droplets. Depending on the flow velocity of the continuous phase, monomer droplets of a well-defined size are ripped off and the continuous formation of monodispersed droplets becomes accessible by the so-called dripping mode (see Video S1). The polymerization and crosslinking process is initiated thereafter by intensive UV irradiation during the transportation of the monomer droplets in the polymerization tube.

To ensure a low viscosity and a sufficient fluidity of the LC-monomer mixture during the injection through the thin glass capillary, the droplet formation was carried out in the isotropic state of the monomer mixture. Therefore, the T-junction and the first part of the polymerization tube was tempered in a heating bath at 110 °C. However, the polymerization of the LC-monomer droplets was carried out in the liquid crystalline state of the monomer mixture, which enabled the formation of a preferred director orientation of the mesogens during the polymerization. For this reason, the monomer droplets were cooled down from the isotropic state to the nematic phase by transportation over a hot plate at 40 °C, at which the UV initiation of the radical polymerization was carried out. Syringe pumps were connected to the microreactor via PTFE microtubes to allow the continuous injection of both the silicone oil and the LC-monomer mixture.

The size of the dispersed LC-monomer droplets and consequently the size of the LCE particles can be controlled by adjusting the ratio of the continuous phase’s flow rate to the dispersed phase’s flow rate. All LCE particles were synthesized at a constant flow rate of the LC-monomer mixture of 0.05 mL/h. Due to the low Reynold numbers occurring under the microfluidic conditions in the presented microreactor [17], laminar flow can be expected to be valid for the LCE particle syntheses.

### 3.3. Adjustment of the Microfluidic Conditions

Previous studies on microfluidic syntheses of actuating LCE particles showed a strong impact of the microfluidic conditions on the particles’ morphology and shape-changing behavior [25]. To investigate the proper adjustment of the microfluidic conditions, the flow rate of the continuous phase was varied to different values. This should lead to conditions at which the actuation properties of the particles exhibit a maximum relative length change and the highest change of the aspect ratio.

Table 1 summarizes the properties of LCE particles, which were synthesized at three different flow rates of the continuous phase, while the crosslinker amount was kept constant at 40 mol %. The calculated values of the particles’ diameter, relative length change and aspect ratio derive from the analysis of at least 15 LCE particles, which were synthesized under the same microfluidic conditions. As expected, the averaged diameter of the LCE particles decreases at higher flow rates of the continuous phase, which is due to the increasing shear rate acting on the droplets at the tip of the capillary. Furthermore, the rapidly produced LCE particles exhibit monodispersity at the three different adjusted flow rates, which means the coefficient of variation of the particles’ diameter is below 5%.

The relative length changes of the particles’ main axis during the phase transition and the aspect ratio of the particles at 30 and 250 °C are also listed in Table 1 for different flow rates of the continuous phase during the polymerization. The particles produced at 1.9 mL/h feature the strongest relative length change as well as the highest increase of the aspect ratio during the phase transition. This suggests a better orientation of the liquid crystalline director field of the LCE. At lower flow rates (1.5 mL/h) the minor amount of shear applied on the monomer droplets during the polymerization is assumed to cause less efficient alignment of the mesogens, which leads to a decrease of the actuation properties. On the other side, higher flow rates reduce the UV irradiation time in the nematic state of the LC-monomer droplets, since the distance of irradiation inside the polymerization tube remains the same (7 cm). This may have caused an incomplete initiation of the polymerization, a minor conversion of the LC-monomers and/or small amounts of residual non-mesogenic EDT, which explains the slight decrease of the particles’ relative length changes at flow rates of 2.3 mL/h compared to the particles produced at 1.9 mL/h.

For this reason, the following particle syntheses were carried out at the medium flow rate of 1.9 mL/h, to ensure both a sufficient UV irradiation and initiation time and high shear rates acting on the droplets during the polymerization. This consequently represents the microfluidic adjustment, which features the optimized actuation properties of the LCE particles.

### 3.4. Actuation Properties of Liquid Crystalline Elastomer Particles

The actuation properties of the LCE particles should be analyzed in terms of the geometry and the magnitude of the shape change during the phase transition, as well as the reversibility of the actuation process. Therefore, heating experiments of single particles were performed in the temperature range of 30 to 250 °C and monitored via polarized optical microscopy. 

**Figure 3:**
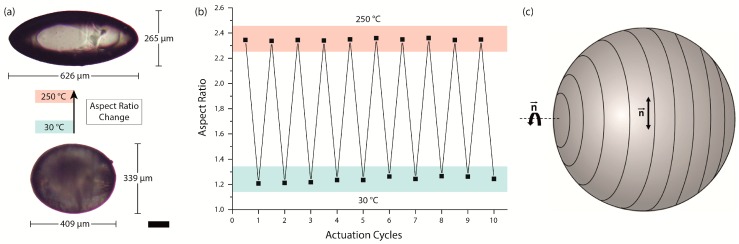
(**a**) Actuation of a single LCE particle before (30 °C, undercooled nematic state) and after (250 °C, isotropic state) the thermal-initiated phase transition. The aspect ratio of the particle is almost doubled during the actuation process; (**b**) Aspect ratio of the particle at 10 sequent actuation cycles, which demonstrates the overall reversibility of the LCE’s shape change; (**c**) Schematic drawing of the supposed concentric director field within the particle. The director is aligned parallel to the black lines (scale bar: 100 µm).

LCE particles showed a shape transformation from a mostly spherical shape to an elongated rod-like shape, at which a particle expands along its main axis and decreases perpendicular to it. This process can be watched in a real-time video of an actuating LCE particle (Video S2), which is placed on a hot stage at 150 °C and cooled down from the isotropic to the undercooled nematic state by cold air streams. This particular actuation pattern has been discovered for LCE particles produced under similar microfluidic conditions before [25]. In this case, a concentric director field orientation (Figure 3c) of the calamitic mesogens is reasonable, as the contraction of the LCE occurs parallel to and the expansion perpendicular to the director of the mesogens.

Figure 3a,b illustrate the actuation process of a single LCE particle, which was synthesized at a flow rate of 1.9 mL/h for the continuous phase, and a crosslinker content of 20 mol %. At 30 °C, the particle’s LCE remains in an undercooled nematic phase, in which slightly colored birefringence can be observed via POM (polarized optical microscopy) using crossed polarizers. Heating up the LCE particle led to the elongation of the particle’s morphology along its main axis and caused an intensification of the colored birefringence, which confirms the nematic state of the LCE. Further heating led to the isotropic state of the LCE at a clearing temperature of 126 °C, at which the particle became completely transparent. Thus, the LC elastomer’s nematic-to-isotropic phase transition temperature could be determined as the temperature by which a loss of birefringence and scattering was observed. At the top of Figure 3a, the particle is illustrated in the isotropic state at 250 °C, featuring the maximum actuation and entire transparency.

During the phase transition, the aspect ratio of this particle is almost doubled from roughly 1.2 to 2.4 and the particle’s main axis exhibits a relative length change of 53%. Compared to previously synthesized thiol-ene–based main-chain LCE particles, which featured relative length changes of up to 23% [35], the actuation properties of the presented particle show strong improvements, with an additional 30% of relative length change. Furthermore, the actuation process is completely reversible during the cooling process of the particle and can be repeated without a significant change of the actuation pattern. Figure 3b demonstrates 10 actuation cycles at which the particle’s aspect ratio was measured alternatively at 30 and 250 °C. During these heating cycles the particle’s aspect ratio offers a coefficient of variation of only 0.3% at high temperatures and 1.7% at low temperatures. The maximum deviation from the mean value of the particle’s aspect ratio reaches 0.5% at 250 °C and 2.4% at 30 °C, which confirms the reversibility of the soft actuator.

### 3.5. Influence of the Crosslinking Density on the Thermomechanical Properties

The actuation properties of LCEs and especially the thermomechanical phase behavior depend on the composition of the liquid crystalline monomer mixture. Since a liquid crystalline crosslinking moiety was used for the LCE particle synthesis, the amount of crosslinker is assumed to dictate the mechanical softness of the material as well as the magnitude of the actuation process [10]. As the LC-crosslinker represents a calamitic mesogen by itself, an influence on the liquid crystalline order and the phase behavior is supposed as well. Therefore, the influence of the crosslinking density on the thermomechanical and actuation properties of the LCE particles should be analyzed.

**Figure 4:**
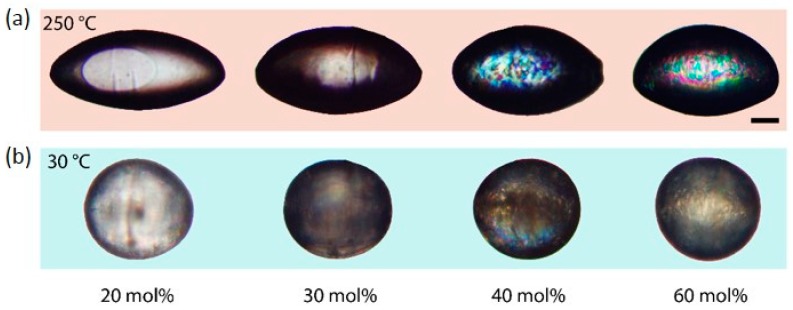
Shape transformation of LCE particles in the temperature range of 30 °C (**b**) and 250 °C (**a**). The particles contain different amounts of the liquid crystalline diacrylate crosslinker and were synthesized under the same microfluidic conditions (images were taken between crossed polarizers, scale bar: 100 µm).

**Figure 5:**
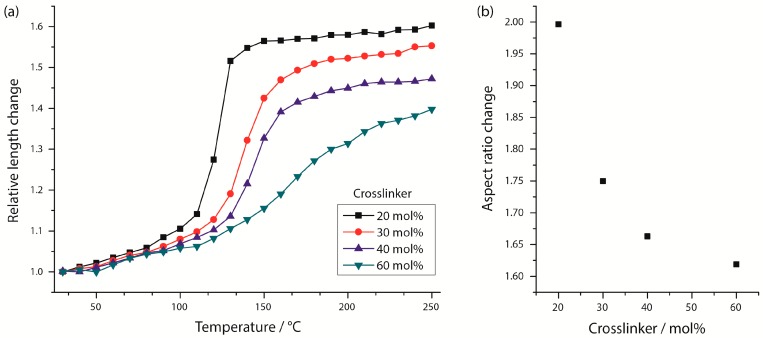
(**a**) Phase behavior of LCE particles, which contain different crosslinker densities. The relative length change along the particles’ long axis l(T)/l(30 °C) is illustrated as a function of the temperature; (**b**) Change of the LCE particles’ aspect ratio AR(250 °C)/AR(30 °C) as dependent on the crosslinker density.

To investigate the impact of the LC-crosslinker content on the phase behavior of the LCE, the crosslinker amount was varied to four different values (20, 30, 40 and 60 mol %), whereas the flow rate of the continuous phase was held constant (1.9 mL/h) during the syntheses. Figure 4 illustrates the shape transformation of LCE particles, which contain different amounts of the LC-crosslinker. The particles exhibit an elongation from a spherical to a rod-like shape by increasing the temperature from 30 to 250 °C as described for the particles in Section 3.4. The phase transition of the LCE and the actuation process occurs in the temperature range of 125 and 220 °C. The relative length changes decrease for an increasing amount of the crosslinking agent. Figure 5b displays the change of the LCE particles’ aspect ratio as a function of the crosslinker amount, which clearly demonstrates the reduction of the intensity of shape change with increasing crosslinking amounts. As was expected, the stiffness of the particles rises for higher crosslinking densities, which restrains the mesogens’ mobility of the LCE material and results in a decrease of the actuation ability [10].

The LCE particles containing 40 and 60 mol % of the LC-crosslinker (top right of Figure 4) show retained birefringence at 250 °C, which indicates a change of the phase behavior as dependent on the crosslinker amount. The behavior of the liquid crystalline phase transition was observed by analysis of the macroscopic shape change of the LCE particles, which correlates with the nematic order parameter *S* [8]. The relative length changes along the main axis of the LCE particles containing different crosslinker densities is illustrated in Figure 5a as a function of the temperature. For the lowest crosslinker density of 20 mol %, the relative length change occurs in a comparatively narrow temperature range, which suggests the character of a first-order phase transition and a weak nematic ordering for temperatures above the clearing temperature (para-nematic phase), at which the length changes are indeed very low, but still exist. An increase of the crosslinker content to 30 and 40 mol % broadens the temperature range, at which the relative length change and, accordingly, the decrease in the nematic order occur. A change of the phase transition’s character to a second-order phase transition is assumed. Especially for the particles with a high crosslinker content of 60 mol %, the phase transition is stretched over a wide temperature range and the relative length change is described by a flattening curve, without showing a clear inflection point. The nematic phase is no longer distinguishable from the para-nematic phase [8].

Two reasons can be assumed to be responsible for the change of the phase behavior with respect to the crosslinking density [9]. Firstly, a high density of the network points immobilizes the nematic order of the mesogens, which consequently prevents the disorganization of the liquid crystalline order even at higher temperatures. Secondly, the liquid crystalline network can be assumed to exhibit a highly inhomogeneous architecture, including parts with high crosslinker densities and parts with fewer network points. Since these diverse areas feature different local clearing temperatures in the LC network, the phase transition of a macroscopic LC sample is broadened.

## 4. Conclusions

A main-chain liquid crystalline elastomer was used to successfully synthesize strongly actuating microparticles with variable properties of the phase behavior and a narrow size distribution. Thiol-ene click chemistry allowed the UV-initiated polymerization of a liquid crystalline monomer mixture containing a mesogenic diallyl monomer and a mesogenic diacrylate crosslinker. The optimization of a capillary-based co-flow microfluidic device enabled the effective orientation of the liquid crystalline monomer mixture in a—as we assume—concentric director field. This leads to an elongation of the crosslinked LCE particles and a rod-like shape transformation during the thermal-initiated phase transition. Changes of the particle’s aspect ratio of almost 100% are thereby available and the entire reversibility of the LCE’s actuation process was demonstrated over several heating and cooling cycles. Furthermore, the modification of the crosslinking density was used to adjust the particle’s total length change as well as the LCE’s phase behavior, such as the clearing temperature and the temperature range of the phase transition. The produced LCE particles establish the basis for further improvements of the actuation properties as well as structural developments, such as implementation in Janus particles or actuating hybrid materials.

## Figures and Tables

**Table 1:** Properties of liquid crystalline elastomer particles synthesized at different flow rates of the continuous phase. The diameter is averaged over the particles’ long and short axis at room temperature. The relative length change describes the ratio of a particle’s main axis in the actuated and the non-actuated state. The aspect ratio is defined as a particle’s main axis divided by the axis perpendicular to the main axis. (CV: Coefficient of variation).

Flow rate (mL/h)	Diameter (µm)	CV ^1^ (%)	Relative length change	Aspect ratio (30 °C)	Aspect ratio (250 °C)
1.5	373.9	4.1	1.360	1.05	1.57
1.9	355.4	3.7	1.483	0.96	1.60
2.3	343.2	4.3	1.402	1.07	1.64

^1^ Coefficient of variation derived from the averaged diameter measurements of at least 15 particles.

## Supplementary Materials

The following are available online at www.mdpi.com/2073-4360/8/12/410/s1, Video S1: Microfluidic droplet formation, Video S2: Actuation process of the LCE particle in real time.
